# Optical coherence tomography findings in unilateral peripheral cone dysfunction syndrome: a case report

**DOI:** 10.1186/s12886-019-1121-2

**Published:** 2019-05-16

**Authors:** Tetsuya Hasegawa, Soichi Tetsuka, Aya Yamaguchi, Chieko Kobashi, Tomomi Sato, Yoshiaki Tanaka, Akihiro Kakehashi

**Affiliations:** 1 Department of Ophthalmology, Jichi Medical University, Saitama Medical Center, 1-847 Amanuma-cho, Omiya-ku, Saitama, Saitama 330-8503 Japan; 2 Chuo Eye Clinic, 2-25-12 Chuo, Ushiku, Ibaraki 300-1234 Japan

**Keywords:** Cone dystrophy, Ganglion cell complex, Swept-source optical coherence tomography, Peripheral cone dysfunction syndrome, Peripheral cone dystrophy

## Abstract

**Introduction:**

To report a case of unilateral peripheral cone dysfunction syndrome and evaluate the associated clinicopathological changes using swept-source optical coherence tomography (SS-OCT).

**Case presentation:**

A 39-year-old Japanese woman reported a visual field defect of 2-years duration in the right eye. The patient underwent visual field testing, full-field electroretinography (ff-ERG), SS-OCT, and a routine ophthalmologic examination. The best-corrected visual acuity was 20/20 bilaterally. The funduscopy examination was normal bilaterally. Visual field testing showed a relative paracentral scotoma in the right eye. SS-OCT scans showed an unclear interdigitation zone (IZ) throughout the posterior pole except for the foveal zone in the right eye. SS-OCT macular analysis showed thinning of the ganglion cell layer (GCL) and inner plexiform layer (IPL) corresponding to the region of the IZ defect. ff-ERG showed almost normal flash ERGs and normal rod responses bilaterally. The cone response and flicker ERG response were decreased markedly only in the right eye.

**Conclusion:**

To the best of our knowledge, this is the first case report of unilateral peripheral cone dysfunction syndrome in which SS-OCT showed pathological changes in the GCL and IPL. The OCT findings corresponded well to the ERG changes and visual field abnormality. Because foveolar cone photoreceptor cells are connected in a one-to-one correspondence to retinal ganglion cells without connection to the horizontal cells or amacrine cells, the GCL and IPL were not present in the fovea. Based on this analysis, we speculated that the primary lesion of peripheral cone dysfunction syndrome is not in the cone photoreceptor cells but in the horizontal cells and/or amacrine cells. The clinicopathological changes in the ganglion cells and cone photoreceptor cells might be the subsequent pathologies in the horizontal cells in peripheral cone dysfunction syndrome.

## Background

We report a case of unilateral peripheral cone dysfunction syndrome, an extremely rare degenerative disease. Peripheral cone dysfunction syndrome and peripheral cone dystrophy are characterized by cone degeneration, but the foveal cone function is maintained. Kondo et al. first determined the pathogenesis of peripheral cone dystrophy using electrophysiology [1]. Thereafter, some cases of peripheral cone dysfunction syndrome and peripheral cone dystrophy have been reported that were analyzed by electroretinography (ERG) and optical coherence tomography (OCT) [2–7]. However, in those reports the pathological analyses had been performed using spectral-domain OCT (SD-OCT). In the current case, we analyzed the thicknesses of the retinal ganglion cell layer (GCL) and inner plexiform layer (IPL) using the latest generation of OCT and swept-source (SS)-OCT and identified the pathognomonic changes in peripheral cone dysfunction syndrome.

## Case presentation

A 39-year-old Japanese woman reported a visual field defect of 2-years duration in her right eye but denied night or day blindness and photopsia. She had a history of high-grade cervical dysplasia of her uterus and no history of long-term medication use. The family history was unremarkable and the parental marriage was not consanguineous.

The patient provided informed consent before the following examinations were performed: a routine ophthalmologic examination, static visual field testing (Humphrey Field Analyzer 3, Carl Zeiss Meditec, Jena, Germany), dynamic visual field testing (Goldmann perimetry, Haag-Streit, Köniz, Switzerland), color vision testing (Ishihara test, Handaya, Tokyo, Japan), full-field ERG (ff-ERG) (LE-3000, Tomey, Tokyo, Japan), SS-OCT (DRI OCT Triton Plus, Topcon, Tokyo, Japan), and fundus autofluorescence (FAF) (DRI OCT Triton Plus).

**Fig. 1:**
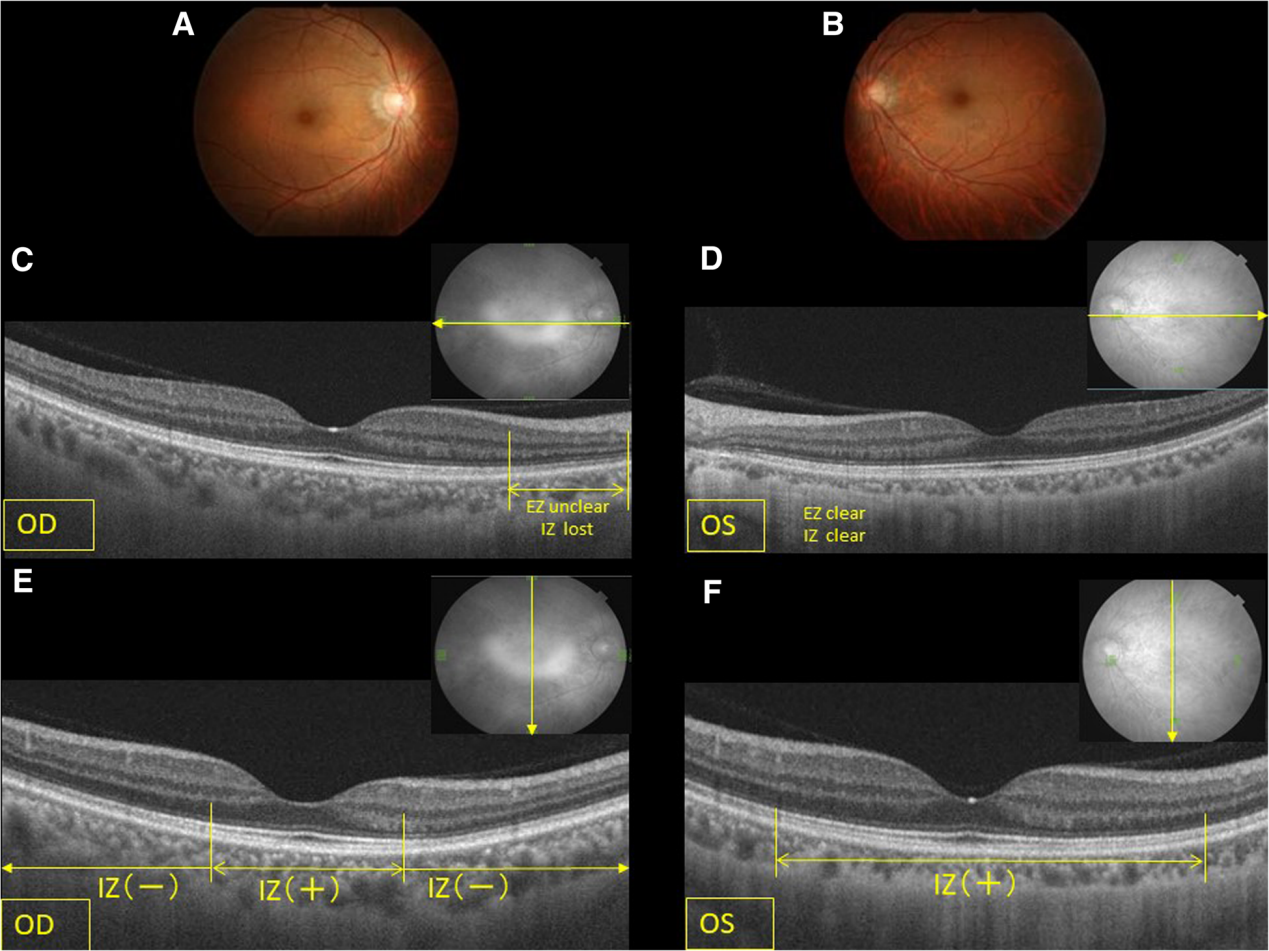
Fundus photographs and swept-source optical coherence tomography horizontal and vertical scans. ***a****:* The fundus photograph of the right eye appears normal. ***b****:* The left eye. ***c****:* Only the horizontal scan of the right eye shows that the ellipsoidal zone (EZ) and interdigitation zone (IZ) are unclear from the parafovea or perifovea to the optic disc. ***d****:* The horizontal image of the left eye appears normal. ***e****:* Only the vertical scan image of the right eye shows that the EZ and IZ are absent from the parafovea or perifovea to the optic disc. ***f****:* The vertical image of the left eye appears normal. OS, left eye; OD, right eye

**Fig. 2:**
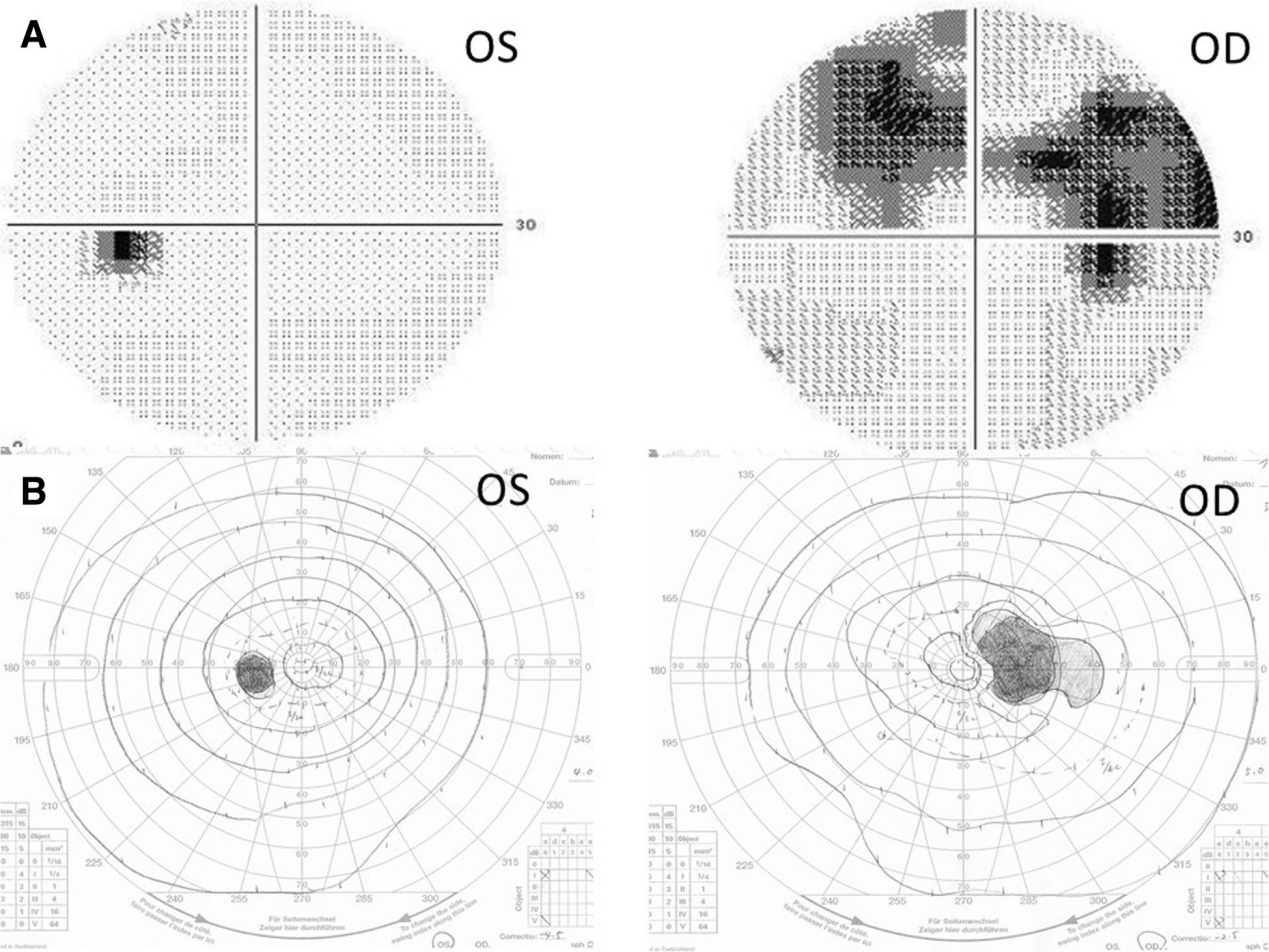
Static and dynamic visual field test results from the current case. ***a****:* Static visual field testing shows a relative paracentral scotoma with central sparing in the right eye. ***b****:* Dynamic visual field testing shows a scotoma, including the Mariotte blind spot, except for the central visual field. OS, left eye; OD, right eye

The best-corrected visual acuity was 20/20 bilaterally with the spherical equivalent of − 4.0 diopters (D) in the right eye and − 7.0 D in the left eye. Anterior segment and funduscopic examinations showed no abnormalities bilaterally (Fig. 1a, b). Color vision testing and FAF of each eye showed no abnormalities. Static visual field test showed a relative paracentral scotoma with central sparing in the right eye (Fig. 2a). Dynamic visual field testing showed the scotoma, including the Mariotte blind spot, except for the central visual field (Fig. 2b).

**Fig. 3:**
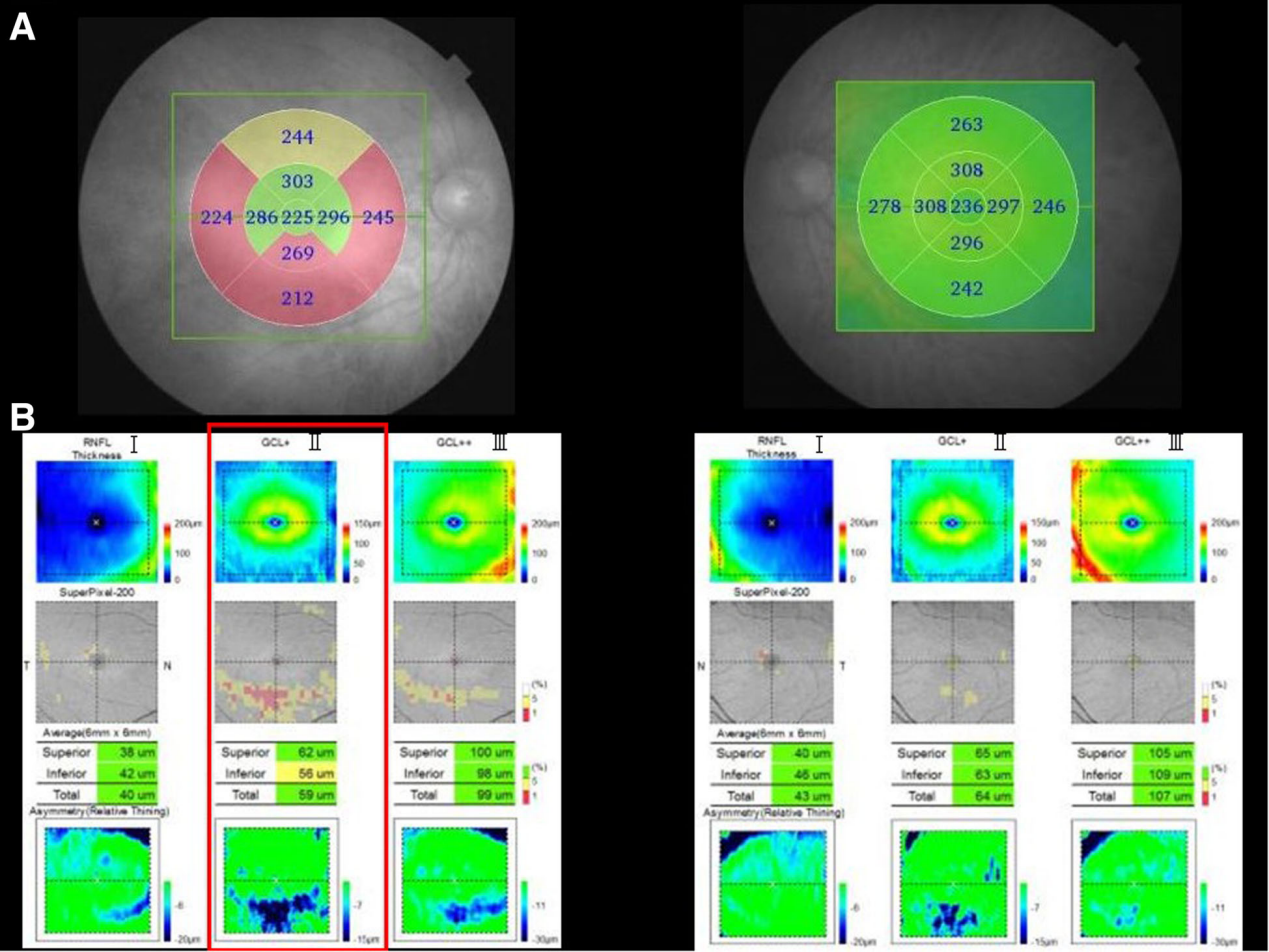
The macular thickness and ganglion cell complex in the current case. ***a****:* The horizontal three-dimensional (3D) macular analysis by swept-source optical coherence tomography (SS-OCT) shows retinal thinning in the parafoveal inferior area and the perifoveal nasal, inferior, and temporal areas in the right but not the left eye. The vertical 3D macular SS-OCT analysis shows thinning of the ganglion cell layer (GCL) and inner plexiform layer (IPL) corresponding to the area of the interdigitation zone defect only in the right eye. ***b****:* In the left eye, all the parameters are normal. I, The retinal nerve fiber layer (RNFL); II, GCL+: the retinal GCL and IPL; III, GCL++: the GCL + IPL + RNFL

The horizontal three-dimensional (3D) macular analysis by SS-OCT showed retinal thinning in the parafoveal inferior area and perifoveal nasal, inferior, and temporal areas in the right eye but no thinning of the macular retina in the left eye (Fig. 3a, b). The horizontal SS-OCT scans showed an unclear interdigitation zone (IZ) throughout the posterior pole except for the foveal zone in the right eye (Fig. 1c). The vertical SS-OCT scans showed that the IZ was absent throughout the posterior pole except for the foveal zone in the right eye (Fig. 1e). The vertical 3D macular SS-OCT analysis showed thinning of the GCL and IPL corresponding to the region of the IZ defect only in the right eye (Fig. 3b). The choroid in the left eye also was thinner compared with the right eye due to high myopia.

**Fig. 4:**
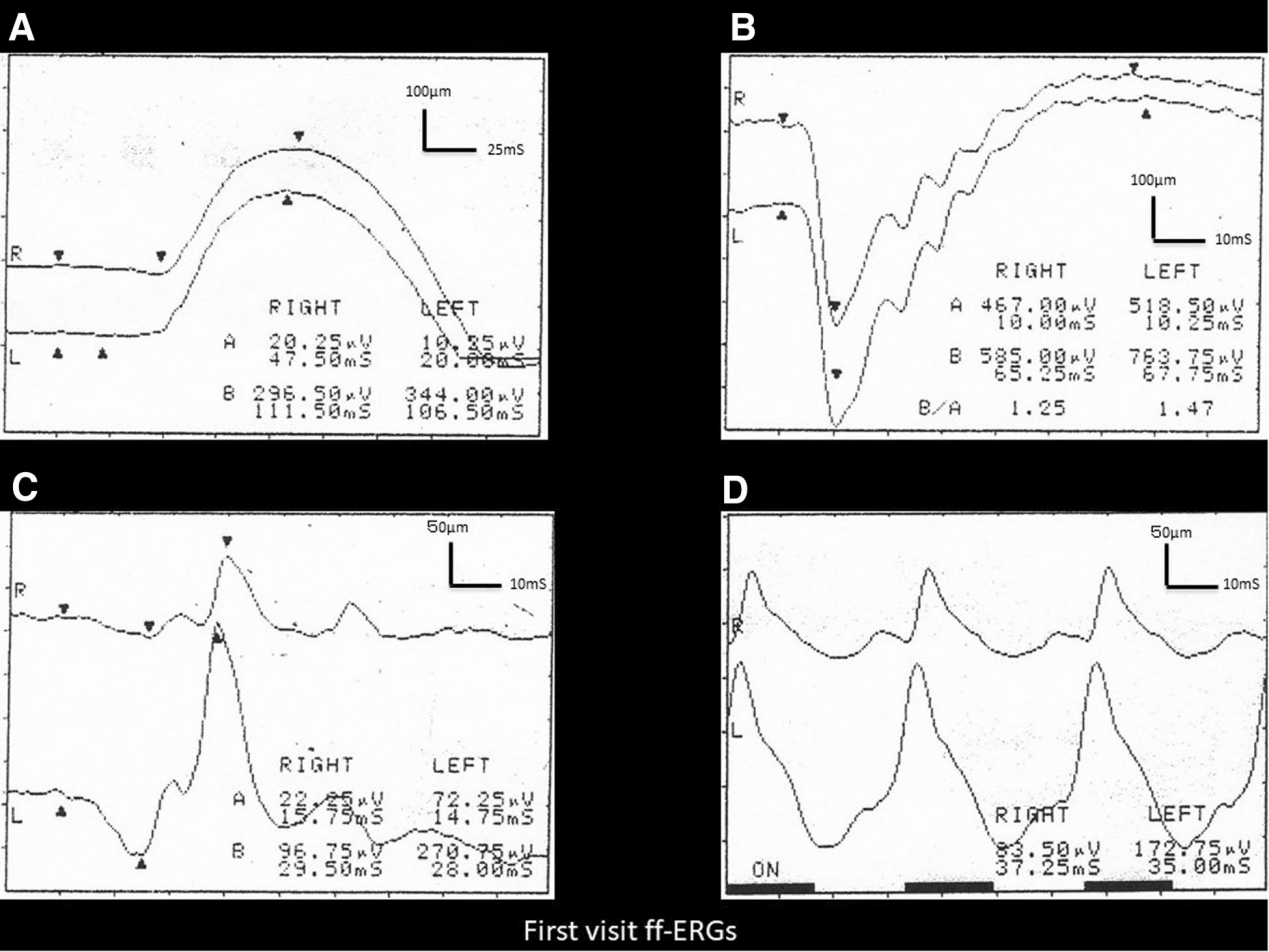
Full-field electroretinography (ff-ERG) images from the current case. ***a****:* The ff-ERG shows almost normal rod responses bilaterally. ***b****:* The single-flash ERG responses are somewhat lower in the right eye. ***c, d****:* The cone response and flicker ERG are decreased markedly only in the right eye. R, right eye; L, left eye

The ff-ERG showed almost normal rod responses bilaterally (Fig. 4a). The single-flash ERG responses were somewhat lower in the right eye (Fig. 4b). However, the cone response and flicker ERG were decreased markedly only in the right eye (Fig. 4c, d). We regularly examined this patient using ERG for 6 years after the first visit. The cone responses decreased by 40.8% and the flicker ERGs decreased by 55% at the final visit compared to the initial ERG recording of 100%. However, the ERG of the left eye was unchanged.

Based on this multimodal analysis, we diagnosed this case as unilateral peripheral cone dysfunction syndrome.

## Discussion and conclusions

To the best of our knowledge, this is the first case report of unilateral peripheral cone dysfunction syndrome in which SS-OCT showed pathological changes in the GCL and IPL. SS-OCT is a significant improvement over conventional SD-OCT due to the optimized long-wavelength scanning light (1050 nm) that facilitates better penetration of the deeper ocular layers, which facilitates the ability to obtain high-quality images from the vitreous to the choroid. Further, a macular thickness map and normal database values for the retinal thickness are available in this model. Thickness maps of the retinal nerve fiber layer (RNFL), GCL + IPL (GCL+), and RNFL+GCL + IPL (GCL++) are also available (Fig. 3b).

When patients report visual field defects without abnormal fundus changes and visual loss, the differential diagnosis includes diseases such as malingering, amblyopia, occult macular dystrophy (Miyake’s disease), retinitis pigmentosa sine pigmento, congenital stationary night blindness (CSNB), cancer-associated retinopathy (CAR), acute zonal occult retinopathy (AZOOR), early cone dystrophy, unilateral peripheral cone dystrophy, and unilateral cone dysfunction syndrome.

In cases of malingering and amblyopia, all ERG responses are normal, and these diagnoses were excluded in the current case. Miyake’s disease is not characterized by an abnormality in the ff-ERG, but the multifocal ERG (mf-ERG) shows an abnormal macular response, and OCT shows attenuation of the ellipsoidal zone (EZ) and IZ in the fovea. Therefore, we excluded Miyake’s disease as a diagnosis in the current case based on the ERG and OCT findings.

In retinitis pigmentosa sine pigmento, CSNB, and CAR, the rod responses are decreased, and these diseases also were excluded from the differential diagnosis.

Patients with AZOOR usually complain of sudden visual field loss accompanied by photopsia. This disease develops in young women and is often unilateral. OCT images show that the EZ and IZ corresponding to the visual field defect are impaired. The ERG shows that all reactions are normal to subnormal. The cone responses tend to be impaired compared with the rod responses. FAF using scanning laser ophthalmoscope shows hyperfluorescence around the optic nerve disc [8]. In the current case, AZOOR was suspected strongly based on the medical history but was excluded because photopsia was absent, the IZ was impaired selectively in the SS-OCT images, the FAF was normal, and the cone response was impaired significantly in the ff-ERG. In localized impairment as indicated by the visual field, the cone response should be unaffected. However, in the current case, the cone response decreased markedly, which reflected widespread cone dysfunction.

Peripheral cone dystrophy has been reported in families [1, 9]. Although the current case seemed to be a genetic disease such as a macular dystrophy, the family history could not be obtained and the changes were unilateral, so this particular case would not be a case of hereditary macular dystrophy. The possibility of drug-induced cone dysfunction syndrome such as that caused by chloroquine [10] and dioxin [11] has been reported. However, there was no history of long-term medication use in the current case, which eliminated drug-induced cone dysfunction syndrome.

In early cone dystrophy, as in the current case, the cone response is decreased or lost in the ff-ERG. The foveal bulge is absent on OCT images. However, in the current case, the foveal bulge was maintained despite loss of the peripheral IZ. Therefore, the findings suggested that this case differed from typical cone dystrophy at the present time. Five cases of unilateral cone dystrophy have been reported [12–15], but the current case is not in the same category because of the residual foveal bulge.

Previous studies have reported the electrophysiologic and clinicopathological findings in peripheral cone dystrophy [1–7]. The ff-ERG of peripheral cone dystrophy shows attenuation or disappearance of the cone response similar to typical cone dystrophy [1, 4–7]. Further, peripheral cone dystrophy is characterized by a normal response at the focal macular ERG and a central response in the mf-ERG [1]. Using FAF, Vaphiades and Doyle reported hyperfluorescence in cases of unilateral peripheral cone dystrophy [4]. However, in the current case, it was normal as reported by Yamada et al. [7]. Mochizuki et al. [3] reported thinning of the outer nuclear layer (ONL), EZ, and IZ using SD-OCT in peripheral cone dysfunction. In the current case, the ONL thickness was not measured, but the disappearance of the IZ in a region other than the fovea occurred only in the right eye. Furthermore, 3D macular SS-OCT analysis showed thinning of the GCL and IPL that corresponded to the area of the IZ defect with foveal sparing only in the right eye. SS-OCT findings corresponded well to the changes in the ERG and visual field abnormality.

Based on this analysis, we speculated that the primary lesion in peripheral cone dysfunction syndrome is not in the cone photoreceptor cells but in the horizontal cells and/or amacrine cells, in that cone photoreceptor cells in the foveola are connected to the retinal ganglion cells in a one-to-one correspondence without connection to the horizontal cells or amacrine cells because the GCL and IPL are not present in the foveal area. The clinicopathological changes in the ganglion cells and cone photoreceptor cells might be secondary changes due to pathology in the horizontal cells and/or amacrine cells in peripheral cone dystrophy. However, the oscillatory potentials (OP) remain in the ERG recording (Fig. 4a). Therefore, the amacrine cells might not be the lesion in the current case, because the amacrine cells are the source of the OP. Thus, we speculated that the main lesions in peripheral cone dystrophy are the horizontal cells. Sieving previously measured the long-flash cone response and suggested that the bipolar cells and/or horizontal cells were the primary lesions [12], which supports our speculation about the pathology in the current case.

The limitation of this case report was the inability to establish a definitive diagnose and clarify the pathophysiology of peripheral cone dysfunction syndrome. First, neither mf-ERG nor focal ERG images, which would be helpful to obtain a definitive diagnose of peripheral cone dysfunction syndrome, were examined. However, the performance of OCT, especially SS-OCT, has improved greatly. By combining the results of the ff-ERG, static visual field test using the Humphrey Field Analyzer, and SS-OCT, it would be possible to establish the clinical diagnosis of a peripheral cone dysfunction syndrome. Second, because long-flash ERG is not available in our institution, we could not evaluate the function of the retinal feedback circuitry. However, theoretically, it is possible to speculate that the main lesions in peripheral cone dystrophy are the horizontal cells based on the results of the examinations in this case.

As Vaphiades and Doyle [4] pointed out, peripheral cone dystrophy is often misdiagnosed. Cases in which patients report a lateral focal visual field abnormality with normal fundus findings and good vision often can be misdiagnosed or left untreated. However, the pathological mechanism of peripheral cone dystrophy can be analyzed by a combination of electrophysiologic examinations and recently developed SS-OCT as in the current case. However, the exact pathogenesis of peripheral cone dystrophy remains uncertain. In addition, no cases of peripheral cone dysfunction syndrome have been followed over the long term; thus, the natural history of this disease remains unknown. It is necessary to follow patients over the long term by performing ophthalmic examinations to check the progress of the lesion.

## Abbreviations

AZOOR: Acute zonal occult retinopathy
CAR: Cancer-associated retinopathy
CSNB: Congenital stationary night blindness
D: Diopters
ERG: Electroretinography
FAF: Fundus autofluorescence
ff-ERG: Full-field electroretinography
GCL: Ganglion cell layer
IPL: Inner plexiform layer
IZ: Interdigitation zone
mf-ERG: Multifocal ERG
OCT: Optical coherence tomography
ONL: Outer nuclear layer
OP: Oscillatory potentials
RNFL: Retinal nerve fiber layer
SD-OCT: Spectral-domain OCT
SS-OCT: Swept-source optical coherence tomography;
